# Ketamine Reduces the Surface Density of the Astroglial Kir4.1 Channel and Inhibits Voltage-Activated Currents in a Manner Similar to the Action of Ba^2+^ on K^+^ Currents

**DOI:** 10.3390/cells12101360

**Published:** 2023-05-10

**Authors:** Mićo Božić, Samo Pirnat, Katja Fink, Maja Potokar, Marko Kreft, Robert Zorec, Matjaž Stenovec

**Affiliations:** 1 Laboratory of Neuroendocrinology-Molecular Cell Physiology, Institute of Pathophysiology, Faculty of Medicine, University of Ljubljana, Zaloška 4, 1000 Ljubljana, Slovenia; 2 Department of Medical Oncology, Institute of Oncology Ljubljana, Zaloška 2, 1000 Ljubljana, Slovenia; 3 Celica Biomedical, Tehnološki Park 24, 1000 Ljubljana, Slovenia; 4 Department of Biology, Biotechnical Faculty, University of Ljubljana, 1000 Ljubljana, Slovenia

**Keywords:** astroglia, ketamine, potassium homeostasis, Kir4.1, vesicle mobility, cAMP, membrane current

## Abstract

A single sub-anesthetic dose of ketamine evokes rapid and long-lasting beneficial effects in patients with a major depressive disorder. However, the mechanisms underlying this effect are unknown. It has been proposed that astrocyte dysregulation of extracellular K^+^ concentration ([K^+^]_o_) alters neuronal excitability, thus contributing to depression. We examined how ketamine affects inwardly rectifying K^+^ channel Kir4.1, the principal regulator of K^+^ buffering and neuronal excitability in the brain. Cultured rat cortical astrocytes were transfected with plasmid-encoding fluorescently tagged Kir4.1 (Kir4.1-EGFP) to monitor the mobility of Kir4.1-EGFP vesicles at rest and after ketamine treatment (2.5 or 25 µM). Short-term (30 min) ketamine treatment reduced the mobility of Kir4.1-EGFP vesicles compared with the vehicle-treated controls (*p* < 0.05). Astrocyte treatment (24 h) with dbcAMP (dibutyryl cyclic adenosine 5′-monophosphate, 1 mM) or [K^+^]_o_ (15 mM), which increases intracellular cAMP, mimicked the ketamine-evoked reduction of mobility. Live cell immunolabelling and patch-clamp measurements in cultured mouse astrocytes revealed that short-term ketamine treatment reduced the surface density of Kir4.1 and inhibited voltage-activated currents similar to Ba^2+^ (300 µM), a Kir4.1 blocker. Thus, ketamine attenuates Kir4.1 vesicle mobility, likely via a cAMP-dependent mechanism, reduces Kir4.1 surface density, and inhibits voltage-activated currents similar to Ba^2+^, known to block Kir4.1 channels.

## 1. Introduction

Astrocytes are abundant neuroglial cells in the central nervous system (CNS) that provide for homeostasis, support survival and function of neurons [1,2], secrete gliosignaling molecules [3,4] and factors that regulate synaptic activity, essential for learning and memory formation [5,6]. During intense neuronal activity, the extracellular concentration of K^+^ ([K^+^]_o_) increases locally and is thought to be cleared by unidirectional redistribution through the astrocyte network (syncytium) toward regions of low K^+^ such as blood vessels. Astroglial K^+^ spatial buffering thus regulates the extracellular concentration of K^+^ ([K^+^]_o_) and controls neuronal excitability in the CNS [7,8,9].

Relatively large astroglial membrane permeability for K^+^ originates from the expression of diverse K^+^ channels including the inwardly rectifying K^+^ channel, Kir4.1 [10,11]. Several studies (reviewed by Nwaobi et al. [12]) including those in vivo [13] have repeatedly confirmed the role of Kir4.1 channels in the regulation of [K^+^]_o_ [14], which may involve isopotentiality through astrocytes connected by gap junctions [9].

Increased awareness of the importance of astrocytes for brain function in health has led to renewed interest in astrocytes as players in the pathogenesis of psychiatric disorders [15,16,17,18,19]. Astrocytes maintain the environment for neuronal networks including the provision of growth factors, therefore, their deficiency may destabilize neural circuits in the brain areas involved in mood regulation. Neuronal activity in the mesolimbic system promotes reward-seeking behavior that results in pleasurable outcomes, whereas neuronal activity in the lateral habenula (LHb) encodes information related to noxious outcomes and suppresses reward-seeking behavior [20]. It is considered that an imbalance between the two systems affects mood states, whereby LHb hyperactivity contributes to major depressive disorder (MDD) [21,22]. One of the mechanisms contributing to the deranged balance between the two systems is modified astroglial K^+^ buffering, which has an impact on neuronal excitability. In the rat LHb, a distinct pattern of neuronal burst firing activity was demonstrated to be linked to reduced [K^+^]_o_, which drives depression [21]. In this model of depression, astroglial Kir4.1 was found upregulated at the transcript, protein, and functional levels [21]. Overexpressed Kir4.1 lowered [K^+^]_o_, hyperpolarizing the membrane potential and causing the burst firing of LHb neurons, resulting in a depression-like phenotype mimicked by lowered [K^+^]_o_. Conversely, the pharmacological blockade or disruption of Kir4.1 function depolarized the membrane potential and caused tonic firing of LHb neurons, resulting in a reduced depression-like phenotype [22]. Ketamine infusion into LHb blocked burst neuronal activity and caused a rapid antidepressant effect [22]. These studies [21,22] provide important insights on how altered K^+^ homeostasis may affect depression-associated LHb neuronal firing, but none of them examined whether ketamine also affected the intracellular transport of Kir4.1 vesicles, altering the surface density of Kir4.1, or the possibility that ketamine may modulate astroglial membrane conductance involving Kir4.1 channels that are broadly expressed in the cortical as well as sub-cortical brain regions [23].

Ketamine, a dissociative anesthetic producing hallucinations, disturbances in thinking and perception, and in motor function [24], has aroused substantial interest amongst psychiatrists [25], since clinical studies have demonstrated that intravenous administration of a single sub-anesthetic dose triggers a rapid and sustained antidepressant response in patients with treatment-resistant major depressive disorder [26,27] and bipolar depression [28]. The rapid antidepressant effect of ketamine contrasts the action of classic antidepressants affecting the monoamine system (i.e., selective serotonin reuptake inhibitors, which elevate the amount of serotonin in the brain), which typically requires weeks to exert an effect. The challenge is thus to understand the mechanisms of the rapid antidepressant action of ketamine versus the delayed action of classic antidepressant drugs. Not only neurons and their glutamatergic NMDA receptors as ketamine targets [29], but also astrocytes have been considered a target of ketamine, where pleiotropic ketamine-mediated functional alterations have been reported including the inhibition of the vesicular secretion of peptides [30], very likely through the ketamine-mediated stabilization of the exocytotic fusion pore in a narrow configuration [31]. Moreover, sub-anesthetic doses of ketamine induce a rapid increase in cytosolic cAMP and an astrocyte-specific remodeling of the plasmalemmal cholesterol composition, a new mechanism of ketamine action [32]. Consistent with this antidepressant treatment causes translocation of the Gα_s_ protein from lipid rafts to non-raft membrane regions [33], likely mediating the changes in cAMP signaling and contributing to the rapid clinical antidepressant effects [25,26,27].

Here, we describe the results revealing that ketamine treatment attenuates the mobility of astrocytic Kir4.1-carrying vesicles, which may contribute to the reduced density of Kir4.1 channels observed at the plasma membrane. Ketamine treatment strongly attenuated voltage-gated currents similar to Ba^2+^, a blocker of Kir4.1 channels in astrocytes. We conclude that ketamine exerts direct effects on the regulation of Kir4.1 channels in isolated astrocytes, contributing to the understanding of the mechanism of action of ketamine in MDD.

## 2. Materials and Methods

### 2.1. Cell Cultures

Primary astrocyte cultures were prepared from neocortices of 2- to 3-day-old female Wistar rats or C57BL/6 mice (obtained from Medical Experimental Center at the Institute of Pathology, University of Ljubljana, Slovenia), as described previously [34]. Animal handling was in accordance with European and Slovenian legislation (Official Gazette of the RS 38/13 and official consolidated text 21/18, 92/20, 159/21; UVHVVR, no. U34401-27/2020/6, U34401-26/2020/4). Briefly, isolated cells were grown in DMEM+ medium (high-glucose (25 mM) Dulbecco’s modified Eagle’s medium [DMEM] supplemented with 10% fetal bovine serum, 1 mM sodium pyruvate, 2 mM L-glutamine, and 5 U/mL penicillin, 5 μg/mL streptomycin) in an atmosphere of 5% CO_2_/95% air until they reached sub-confluent density. Then, after three consecutive shaking cycles (at 225 rpm overnight with subsequent replacement of medium), purified astrocytes were sub-cultured in tissue culture tubes. Before the experiments, cells were trypsinized, plated (50 μL of cell suspension) onto poly-L-lysine (PLL)-coated coverslips, and left for 20 min at 37 °C in an atmosphere of 5% CO_2_/95% air to allow cells to attach to the coverslips, which were further maintained in the growth medium. Unless stated otherwise, all chemicals were purchased from Merck (Darmstadt, Germany) and were of the highest grade purity available.

### 2.2. Solutions

Extracellular bath solution (ECS) consisted of 130 mM NaCl, 5 mM KCl, 2 mM CaCl_2_, 1 mM MgCl_2_, 10 mM D-glucose, and 10 mM HEPES/NaOH (pH 7.4). High [K^+^] extracellular solution consisted of 60 mM NaCl, 75 mM KCl, 2 mM CaCl_2_, 1 mM MgCl_2_, 10 mM D-glucose, and 10 mM HEPES/NaOH (pH 7.4). The osmolarity of the solutions (300 ± 15 mOsm) was measured with a freezing-point osmometer (Osmomat 030, Gonotec, Berlin, Germany). Ketamine hydrochloride (KM; Tocris Bioscience, Bristol, UK) was added to the culture medium 30 min before the start of the experiments to reach a final concentration of 2.5 or 25 µM; dibutyryl cAMP (dbcAMP, Merck) or KCl was added to the culture medium 24 h before the start of the experiments to reach a final concentration of 1 mM or 15 mM, respectively. During the experiments, all agents (KM, dbcAMP and high [K^+^]) were also provided in equal concentration in ECS. For acute application, high [K^+^] extracellular solution was added to the ECS as a bolus to reach a final concentration of 15 mM K^+^.

For the electrophysiology, astrocytes were bathed in ECS containing 138 mM NaCl, 5 mM KCl, 2 mM CaCl_2_, 1.9 mM MgCl_2_, 10 mM HEPES, 3 mM tetraethylammonium chloride (TEACl), 6 mM 4-aminopyridine/HCl, pH 7.4 adjusted with NaOH. Fire-polished, standard-walled borosilicate glass pipettes (30-0058, Harvard Apparatus, Holliston, MA, USA) were filled with a solution containing 130 mM KCl, 1 mM CaCl_2_, 1 mM MgCl_2_, 10 mM HEPES, 2 mM Na_2_ATP, 10 mM EGTA, 0.25 mM spermine/HCl, pH 7.2 adjusted with KOH. The osmolarity of both solutions (300 ± 10 mOsm) was measured with a freezing-point osmometer (Osmomat 030, Gonotec, Berlin, Germany). All recordings were performed at room temperature (RT). Stimuli were added to the recording chamber as a bolus (100 µL) to reach a final concentration of 300 µM Ba^2+^, 2.5 µM ketamine, or a mixture of both in the ECS.

### 2.3. Plasmid and Cell Transfection

To visualize Kir4.1-positive vesicles, we transfected cultured astrocytes with plasmid-encoding wild-type Kir4.1 tagged with enhanced green fluorescent protein (pKir4.1-EGFP, a gift from Drs. M. Eaton and S. Skatchkov) [35,36]. First, 3 µL of FuGENE 6 (Promega Corporation, Madison, WI, USA) was diluted in 100 µL of culture medium, mixed, and incubated for 5 min at RT. Next, DNA (0.2 µg/vial) was added, mixed, and incubated for a further 15 min at RT. Astrocytes were washed and subsequently incubated in 900 µL of fresh culture medium to which 100 µL of the lipofection mixture was added. Transfected astrocytes were incubated for 24 h at 37 °C in an atmosphere of 5% CO_2_/95% air. The transfection medium was exchanged for fresh culture medium the next day, and transfected astrocytes were observed microscopically after 24–48 h.

### 2.4. LysoTracker Labelling

Acidic late endo-/lysosomes were labelled by incubating transfected cells in culture medium containing 200 nM LysoTracker red DND-99 (LyTr; Thermo Fisher Scientific, Waltham, MA, USA) for 5 min at 37 °C. LysoTracker-labelled cells were washed once with extracellular solution, mounted onto the recording chamber, supplied with ECS, and then observed on a confocal microscope (LSM 780, Zeiss, Jena, Germany).

### 2.5. Fixed and Live Cell Immunocytochemistry and Fluorescence Analysis

To structurally characterize the Kir4.1 vesicles, transfected rat astrocytes were fixed in formaldehyde (4%) for 15 min, permeabilized with 0.1% Triton X-100 for 10 min, and then washed four times with phosphate-buffered saline (PBS), all at RT. Non-specific background staining was reduced by incubating the cells in blocking buffer with 10% (*v*/*v*) goat serum in 3% (*w*/*v*) bovine serum albumin (BSA) in PBS for 1 h at 37 °C. Cells were then washed with PBS once and incubated with primary antibodies diluted in 3% BSA in PBS overnight at 4 °C. The following primary antibodies were used: rabbit polyclonal anti-Kir4.1 (1:500; APC-165, Alomone, Jerusalem, Israel), rabbit polyclonal anti-aquaporin 4 (AQP4; 1:400, sc20812, Santa Cruz Biotechnology, Dallas, TX, USA), mouse polyclonal anti-synaptotagmin IV (SytIV; 1:200, sc30095, Santa Cruz Biotechnology), mouse monoclonal anti-synaptobrevin 2 (Syb2/VAMP2; 1:500, 104211, Synaptic Systems, Goettingen, Germany), rabbit polyclonal anti-VAMP3 (1:1000, Abcam, Cambridge, UK), mouse monoclonal anti-microtubule-associated protein 1 light chain 3 (LC3; 1:100, M152-3, MBL, Woburn, MA, USA), rabbit monoclonal anti-Rab7 (1:400; ab137029, Abcam), mouse monoclonal anti-α-tubulin (1:100–1:200; T-6199, Merck), and mouse monoclonal anti-β-actin (1:500–1:1000; Clone AC-15, A5441, Merck). The next day, the cells were washed four times in PBS and stained with secondary anti-rabbit or anti-mouse antibodies conjugated to Alexa Fluor 546 or 555 (1:800; Thermo Fisher Scientific), respectively, for 45 min at 37 °C, and then washed four times in PBS. The coverslips were then mounted onto glass slides using the SlowFade Gold antifade mountant with or without DAPI (4′,6-diamidino-2-phenylindole; Thermo Fisher Scientific).

To quantify the co-localization of green-emitting fluorophores (Kir4.1-EGFP) and red-emitting fluorophores, tiff files were exported and analyzed with ColocAna (Celica Biomedical, Ljubljana, Slovenia), which enables automated high throughput co-localization analysis of fluorescent markers in a large number of images [37]. Briefly, the program counted all green, red, and co-localized (green and red) pixels in each image. Fluorescence co-localization (%) was determined with reference to green pixels identifying Kir4.1 vesicles. The threshold for the co-localized pixel count was set to 20% of the maximal fluorescence to reduce the apparent fluorescence overlap originating from the closely positioned fluorescent structures. In live cell labelling, the fluorescence co-localization between red-emitting LyTR and green-emitting fluorescent Kir4.1-EGFP was quantified.

Immunocytochemical labelling of live mouse astrocytes was performed similarly to that described previously [38]. Cells were first washed once with 3% BSA in PBS and incubated with rat polyclonal anti-Kir4.1 (clone 20F9, 1:500; a gift from Prof. Dr. Bernhard Hemmer, Technische Universität München, Munich, Germany) diluted in 3% BSA in PBS for 45 min at 37 °C. Subsequently, cells were washed three times with 3% BSA in PBS and incubated with corresponding secondary antibodies for 45 min at 37 °C. Cell-loaded coverslips were then washed three times with 3% BSA in PBS and once with extracellular solution, and transferred to the recording chamber. Immunofluorescent cells were observed with a confocal microscope (LSM 780, Zeiss) with a plan apochromatic oil-immersion objective 63×/NA 1.4. Confocal images (single planes or z stacked) were obtained with 561-nm diode-pumped solid-state laser excitation, and the fluorescence emission was bandpass filtered at 565–615 nm, respectively.

To measure the apparent size (surface area) and cumulative fluorescence intensity of immunofluorescent Kir4.1 at the surface of the astrocytes, confocal images of the live cells were analyzed with ImageJ 1.53t software (available at National Institute of Health, Bethesda, MD, USA, http://rsbweb.nih.gov/ij/ accessed on 8 January 2021). The minimum size of a fluorescent spot taken to identify an individual Kir4.1-positive punctum was three adjacent pixels (0.176 × 0.176 μm), and the minimum surface area covered by a punctum was 0.093 µm^2^. This way, a broad array of puncta with different apparent sizes and intensities was covered by the analysis.

### 2.6. Analysis of Kir4.1 Vesicle Mobility

Coverslips with astrocytes were mounted into the recording chamber and transferred to a confocal microscope (LSM 780; Zeiss) equipped with a plan apochromatic oil-immersion objective 63×/NA 1.4. Kir4.1-EGFP was excited by a 488-nm argon laser line and emission fluorescence was filtered with a bandpass filter (495–545 nm). Time-lapse images were acquired at a rate of 2 Hz for 1 min before and 1, 2, 5, and 25 min after the application of high [K^+^] extracellular solution to the cells. Vesicle mobility was analyzed in exported tiff files with the ParticleTR 2 software (Celica Biomedical) as previously described [39,40]. Typically, 20 mobile vesicles were selected per transfected astrocyte and their movement was tracked automatically as long as they remained in focus in each image plane while moving. Whenever the tracked vesicle moved into close proximity to another vesicle, automatic tracking was halted and the position of the selected vesicle was determined manually in subsequent frames until the tracked vesicle separated enough from the neighboring vesicle to resume automated tracking. The following mobility parameters were obtained for 15 s epochs: TL (track length; total length of the travelled pathway), MD (maximal displacement; the farthest translocation of a vesicle), DI (directionality index; the ratio of MD/TL), and speed [39].

### 2.7. Electrophysiology

Astrocyte-loaded coverslips were placed into the recording chamber, supplied with ECS, and mounted on an inverted microscope (Zeiss IM35). We measured uncompensated macroscopic currents in whole-cell configuration by using fire-polished, standard-walled borosilicate glass pipettes (30-0058, Harvard Apparatus, Holliston, MA, USA) with a resistance of 6 MΩ, at RT, and a dual-phase lock-in patch-clamp amplifier (SWAM IIC, Celica Biomedical, Ljubljana, Slovenia), as described previously [41]. Cells were voltage-clamped at −70 mV and hyperpolarized or depolarized to −90 mV to +10 mV by rectangular pulses (lasting for 100 ms and separated by 50 ms) generated by WinWCP V5.6.2 software (John Dempster, University of Strathclyde, Glasgow, UK; available at: https://spider.science.strath.ac.uk/sipbs/software_ses.htm accessed on 16 November 2021). The clamped voltage and macroscopic currents were recorded with a SWAM IIC amplifier, fed through a digitizer (National Instruments BNC-2111, Austin, TX, USA) and stored in a computer with the WinWCP V5.6.2 data acquisition system. The membrane capacitance was read from the amplifier immediately after attaining the whole-cell configuration at a lock-in frequency of 800 Hz (sine wave of 1.1 mV rms); the latter was switched off during current measurements. In each cell, currents were measured (for 70 ms during the voltage step) before and 1–3 min after bolus addition of pharmacological inhibitors to reach the final concentration of 300 µM Ba^2+^, 2.5 µM ketamine, or a mixture of both. Extracellular application of Ba^2+^ leads to an inhibition of Kir currents in a voltage-dependent manner; the effect on Kir currents is stronger at hyperpolarized voltages with a dissociation constant of Ba^2+^ being independent of the extracellular K^+^ concentration [42]. Current amplitudes were analyzed with a custom-written MATLAB program (MathWorks, Natick, MA, USA). Recordings with an access conductance (G_a_) of <50 nS were excluded from the analysis unless the measured cell was small (C_m_ ≤ 30 pF), and the current recordings were stable throughout. All data obtained in cells displaying currents inhibited by Ba^2+^ and/or ketamine are reported.

### 2.8. Statistical Analysis

All measured parameters are presented as the means ± standard error of the mean (SEM). Statistical significance was determined with the Mann-Whitney U test (electrophysiological measurements) or ANOVA on ranks followed by Dunn’s post hoc test (measurements of vesicle mobility and Kir4.1 surface immunofluorescence) using SigmaPlot 11.0 (Systat Software, San Jose, CA, USA).

## 3. Results

### 3.1. Kir4.1 Localizes to Aquaporin 4-Positive Vesicles and Vesicles Competent for SNARE- and Ca^2+^-Dependent Exocytosis

**Figure 1:**
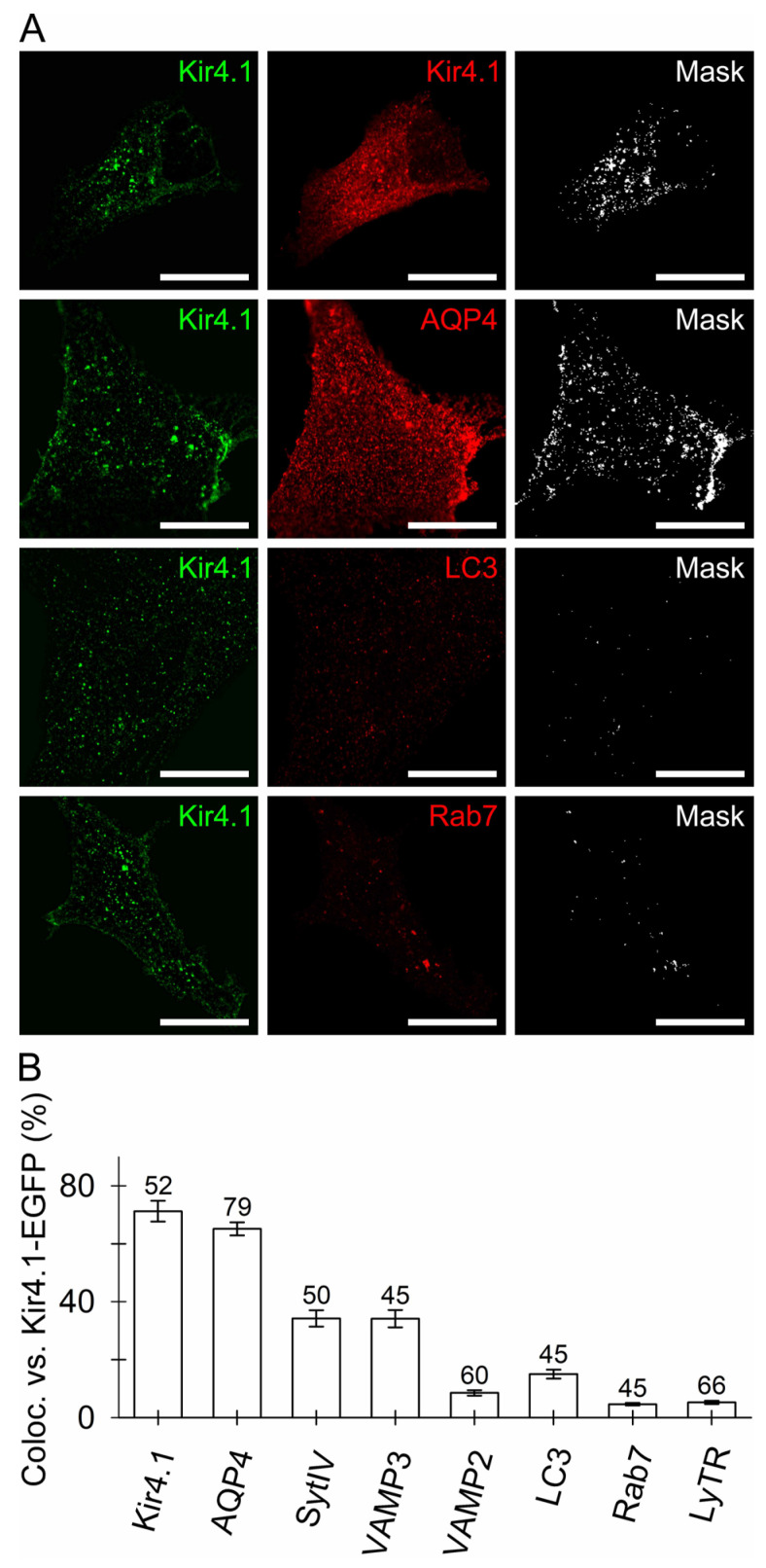
Immunocytochemical characterization of the Kir4.1 vesicles in rat astrocytes. (**A**) Confocal micrographs of fixed transfected astrocytes expressing Kir4.1-EGFP (green, left) labelled with primary antibodies against Kir4.1, aquaporin 4 (AQP4), microtubule-associated protein 1 light chain 3 (LC3), Rab7, a protein characteristic of late endosomes and multivesicular bodies as well as of autophagosomes and lysosomes, and the corresponding Alexa Fluor 546-conjugated secondary antibodies (red, middle). The mask images (white, right) display co-localized pixels. Scale bars: 20 μm. (**B**) Graph displaying quantitative co-localization (%, mean ± SEM) of anti-Kir4.1, anti-AQP4, anti-SytIV, anti-VAMP3, anti-VAMP2, anti-LC3, anti-Rab7, and LyTR fluorescence versus Kir4.1-EGFP fluorescence. The numbers above the bars indicate the number of cells analyzed.

To study the subcellular distribution of Kir4.1, we transfected cultured cortical rat astrocytes with a plasmid to express fluorescently tagged Kir4.1 (Kir4.1-EGFP). Transfection yielded a punctate appearance of green fluorescence in the astrocyte cytoplasm (Figure 1A, left). These puncta exhibited mobility at rest, as previously shown for peptidergic vesicles [43], indicating that Kir4.1-EGFP may be present on the vesicles. We then asked whether the transfection-induced green fluorescent puncta co-localized with the endogenous Kir4.1, which was labelled with a primary anti-Kir4.1 antibody (red fluorescence; Figure 1A, middle). The co-localization mask (Figure 1A, right) revealed that the transfected Kir4.1 channel accounted for 71 ± 4% (mean ± SEM, *n* = 52; Figure 1B) of the endogenous Kir4.1 structures under these experimental conditions. Moreover, it was previously reported that Kir4.1 appears to co-localize with aquaporin 4 [44], a water channel expressed mainly in astrocytes, therefore, we also examined the fluorescence co-localization between Kir4.1-EGFP and the AQP4 structures labelled by a primary antibody. As expected, the Kir4.1-EGFP- and AQP4-positive structures were relatively highly co-localized (65 ± 2%, *n* = 79; Figure 1A, middle and right; Figure 1B). Next, we examined whether Kir4.1-EGFP-positive compartments expressed the SNARE proteins, known to be required for Ca^2+^-dependent exocytosis [45,46]. Kir4.1-EGFP fluorescence was modestly co-localized with the immunostained SNARE-associated protein synaptotagmin IV (SytIV; 34 ± 3%, *n* = 50; Figure 1B), a modulator of the Ca^2+^-dependent exocytotic release of gliosignaling molecules from astrocytes [47], and with VAMP3 (34 ± 3%, *n* = 45) as well as to a minor extent with VAMP2 (8 ± 1%, *n* = 60; Figure 1B), the two vSNARE proteins characteristic of vesicles undergoing regulated exocytosis [45]. These data indicate that a fraction of astroglial Kir4.1-EGFP vesicles may engage in SNARE- and Ca^2+^-dependent exocytosis. The fluorescence co-localization between the Kir4.1-EGFP vesicles and immunolabelled LC3, a protein characteristic of autophagosomes [48], was relatively low (15 ± 2%, *n* = 45; Figure 1B). Moreover, co-localization with Rab7, a protein characteristic of late endosomes and multivesicular bodies [49,50] as well as with autophagosomes or lysosomes [49,51] was also relatively low (5 ± 0%, *n* = 45; Figure 1B), indicating that Kir4.1 is expressed to a small extent in the endo-lysosomal and autophagic compartments under these experimental conditions. This was further confirmed by the relatively low fluorescence co-localization between the Kir4.1-EGFP vesicles and LyTR, a dye accumulating inside acidic organelles [52] (5 ± 1%, *n* = 66; Figure 1B). Collectively, these results indicate that under our experimental conditions, Kir4.1-EGFP predominantly localized to AQP4-positive vesicles with a minute fraction as well as to endo-lysosomes (Figure 1).

### 3.2. Similar Mobility of Kir4.1 Vesicles and Endo-Lysosomes

**Figure 2:**
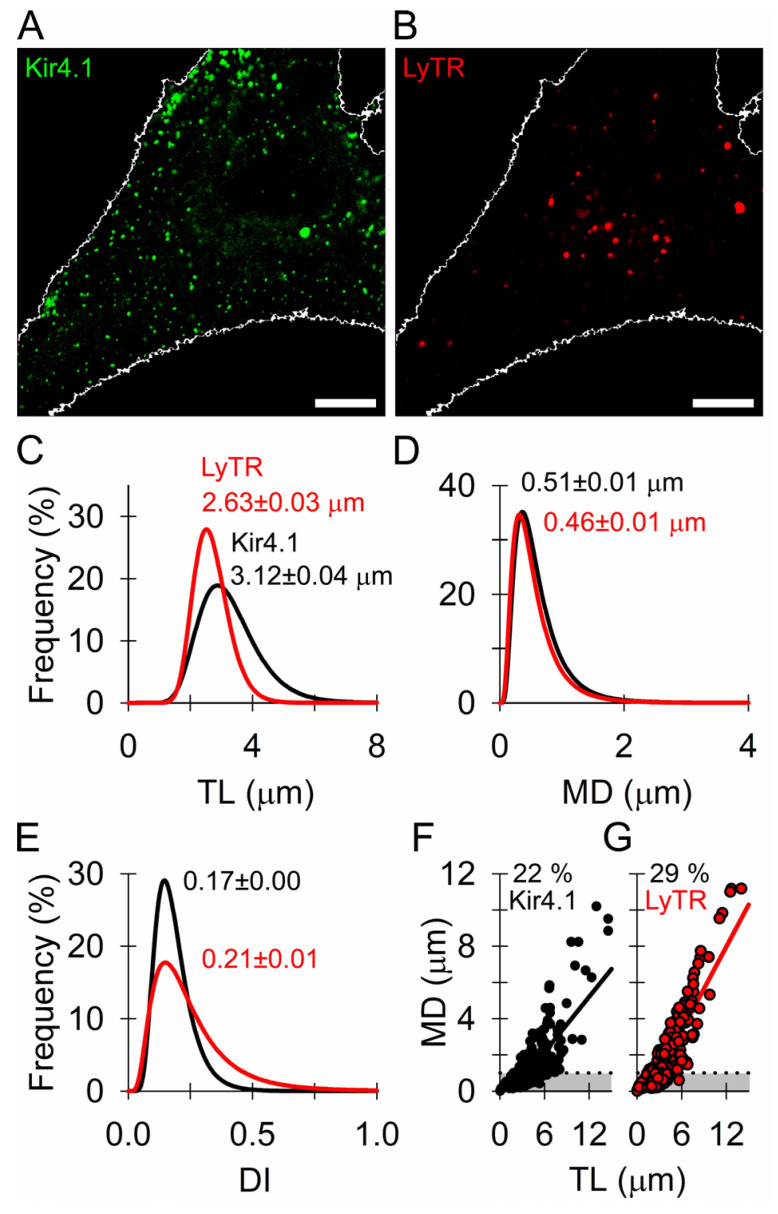
Spontaneous mobility of Kir4.1 vesicles resembles the mobility of endo-lysosomes. (**A**,**B**) Confocal micrographs of Kir4.1 (**A**) and LysoTracker-laden vesicles (LyTR) in the cytosol of the transfected astrocyte (**B**); scale bars, 10 µm. (**C**–**E**) Relative frequency distribution of the track length (TL; (**C**)), maximal displacement (MD, (**D**)), and directionality index (DI; (**E**)) obtained in the Kir4.1 and LyTR-laden vesicles (640 vesicles in 8 cells, respectively) after fitting the data with the logarithmic Gaussian function (Kir4.1, black curve; LyTR, red curve) of the form: *f* = *a* × exp(−0.5(*x*/*x*_0_)/*b*)^2^/*x*, where *a* = 56.63 ± 2.18, *b* = 0.29 ± 0.01 μm^−0.5^, *x*_0_ = 3.12 ± 0.04 μm (Kir4.1) and *a* = 71.87 ± 2.94, *b* = 0.21 ± 0.01 μm^−0.5^, *x*_0_ = 2.63 ± 0.03 μm (LyTR) in TL data (**C**), *a* = 15.21 ± 0.19, *b* = 0.58 ± 0.01 μm^−0.5^, *x*_0_ = 0.51 ± 0.01 μm (Kir4.1) and *a* = 13.23 ± 0.36, *b* = 0.61 ± 0.02 μm^−0.5^, *x*_0_ = 0.46 ± 0.01 μm (LyTR) in the MD data (**D**) and *a* = 4.57 ± 0.20, *b* = 0.39 ± 0.02 μm^−0.5^, *x*_0_ = 0.17 ± 0.00 (Kir4.1) and *a* = 3.13 ± 0.24, *b* = 0.58 ± 0.05 μm^−0.5^, *x*_0_ = 0.21 ± 0.01 (LyTR) in the DI data (**E**). The mean of the Gaussian function (*x*_0_; mean ± SEM) is displayed above each curve. (**F**,**G**) Plots display the relationship between MD and TL in the Kir4.1-positive vesicles ((**F**); black circles) and LyTR-positive vesicles ((**G**); white circles). The percentage of vesicles with MD >1 μm (above the dashed line delimiting the grey area) is shown in panels exhibiting a more directional mode of mobility, likely involving cytoskeletal elements.

To further establish the nature of organelles carrying Kir4.1-EGFP (Figure 1), we examined their mobility and compared that with the mobility of LyTR-laden acidified endo-lysosomes [52] within the same cells (Figure 2A,B). We analyzed 640 mobile Kir4.1-EGFP- and LyTR-positive vesicles in eight cells from two animals. The frequency histograms of individual mobility parameters, fitted with a logarithmic Gaussian function (for details, see legend to Figure 2C–E), revealed that in the Kir4.1-EGFP-positive vesicles (black curve), TL, MD, and DI exhibited mean (±SEM) values of 3.12 ± 0.04 µm (Figure 2C), 0.51 ± 0.01 µm (Figure 2D), and 0.17 ± 0.00 (Figure 2E), respectively. In the LyTR-laden vesicles (red curve), the mean values were 2.63 ± 0.03 µm (Figure 2C), 0.46 ± 0.01 µm (Figure 2D), and 0.21 ± 0.01 (Figure 2E), respectively. These parameters were similar in both vesicle types studied. No single parameter of mobility differed in both vesicles, as revealed by the Kolmogorov–Smirnov test. Next, we examined the relationship between TL and MD in the Kir4.1-EGFP- and LyTR-positive vesicles and estimated the percentage (%) of vesicles that exhibited MD >1 µm within a 15 s epoch. The percentage of vesicles with MD >1 µm was lower in the Kir4.1-EGFP-positive vesicles (142 of 640, 22%; Figure 2F) than in the LyTR-positive vesicles (182 of 638, 29%, *p* < 0.01, χ^2^ test; Figure 2G). These results indicate that the relatively more mobile Kir4.1-EGFP-positive vesicles (22%) may be associated with cytoskeletal elements, as previously reported for peptidergic astrocytic vesicles [53,54].

### 3.3. Kir4.1 Vesicles Associate More with Microtubules Than with Actin Filaments

**Figure 3:**
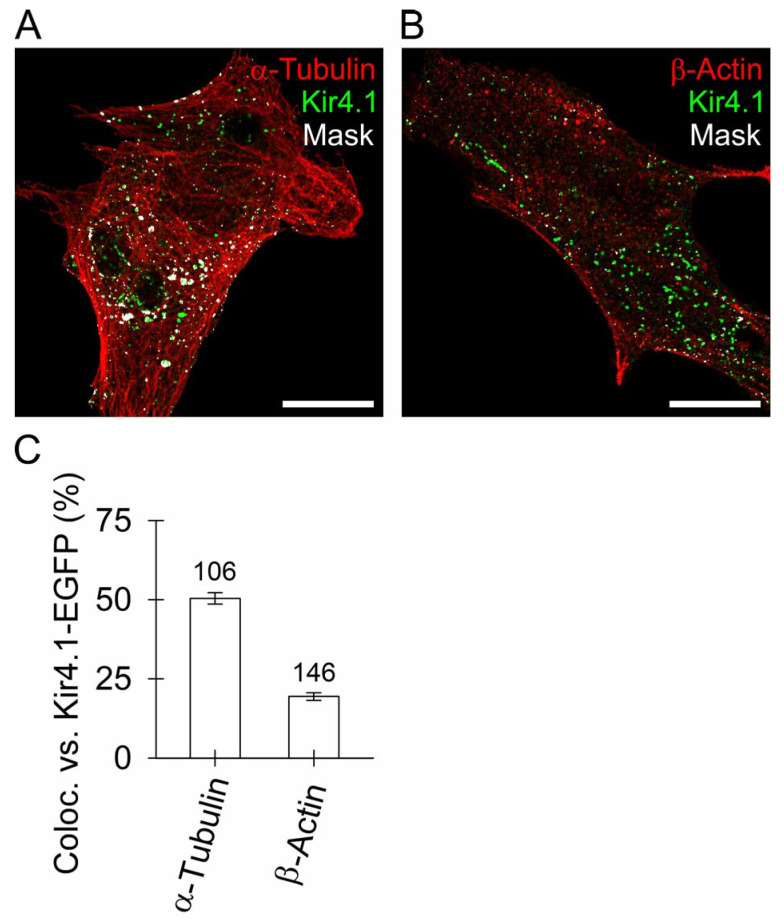
Astroglial Kir4.1 vesicles amply localize in proximity to the microtubules and scarcely along the actin filaments. (**A**,**B**) Double fluorescent confocal images display astroglial Kir4.1-EGFP vesicles (green) and immunofluorescent microtubules (α-tubulin, red, (**A**)) or actin filaments (β-actin, red, (**B**)) in the same cells. Scale bars: 20 μm. Kir4.1 vesicles amply localized along microtubules (188 of 328) and less along the actin filaments (78 out of 295) as shown by the superimposed white co-localization masks. (**C**) Quantitative co-localization (%, mean ± SEM) of Kir4.1-EGFP fluorescence with immunofluorescent microtubules (50.4 ± 1.8%) and actin filaments (18.8 ± 1.3%). The numbers above the bars indicate the number of cells analyzed.

To optophysiologically examine whether Kir4.1-EGFP-positive vesicles structurally associate with cytoskeletal elements, we immunofluorescently labelled microtubules or actin filaments in Kir4.1-EGFP-transfected rat astrocytes. The results shown in Figure 3 display the fluorescence co-localization mask images (white, Figure 3A,B) where Kir4.1-positive vesicles are localized in close proximity to the microtubules (50 ± 2%; Figure 3C), and less along the actin filaments (19 ± 1%; Figure 3C). These data indicate that vesicular interactions with microtubules likely contribute to the directional mobility of Kir4.1 in the cytoplasm, possibly also toward the plasmalemma.

### 3.4. Ketamine and an Increase in Intracellular [cAMP] Attenuate the Mobility of Kir4.1-EGFP-Positive Vesicles

**Figure 4:**
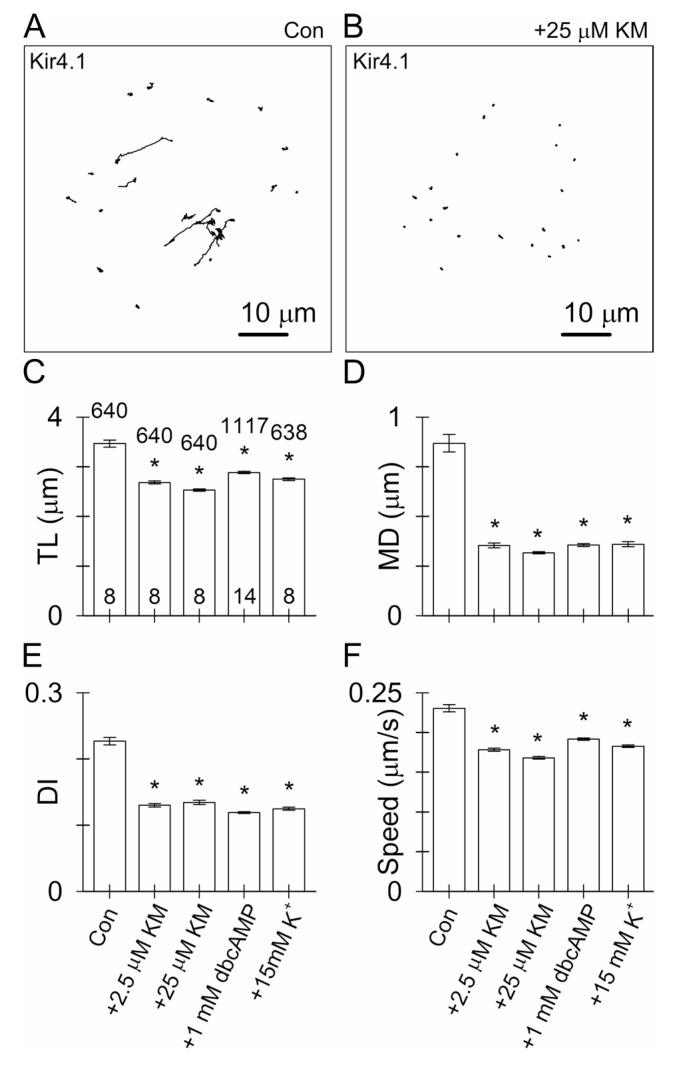
Short-term treatment with ketamine (KM) attenuates the mobility of astroglial Kir4.1-positive vesicles. (**A**,**B**) Reconstructed tracks of Kir4.1 vesicles (*n* = 20, 1-min epoch) in the controls and astrocytes treated for 30 min with 25 µM KM. In the control astrocytes, elongated tracks revealed substantial vesicle mobility, whereas in the KM-treated astrocytes, vesicle mobility was limited as indicated by highly contorted vesicle tracks. (**C**–**F**) Spontaneous mobility of Kir4.1 vesicles (TL, (**C**); MD, (**D**); DI, (**E**) and speed, (**F**); mean ± SEM) in the non-treated controls (Con) and astrocytes treated for 30 min with 2.5 or 25 µM KM or for 24 h with 1 mM dbcAMP or 15 mM K^+^ added to the culture media. Different treatments evoked comparable reductions in the mobility of Kir4.1 vesicles. The numbers at the top and bottom of the bars indicate the number of vesicles (15 s epochs) and the number of cells analyzed, respectively. * *p* < 0.05 versus mobility in the controls (ANOVA on the ranks followed by Dunn’s post hoc test).

Next, we examined how KM, which increases intracellular [cAMP] [32], but not [Ca^2+^]_i_ in astrocytes [30], alters the mobility of Kir4.1-EGFP-positive vesicles. Vesicle mobility was recorded for 1 min in the rat non-treated astrocytes (control) and in astrocytes treated for 30 min (short-term) with 2.5 or 25 µM KM, and in astrocytes treated for 24 h with either 1 mM dbcAMP, a membrane permeable analog of cAMP [55,56], or 15 mM K^+^, which was reported to increase [cAMP]_i_ via bicarbonate-responsive soluble adenylyl cyclase in cultured rat astrocytes and hippocampal slices [57,58], but did not increase [Ca^2+^]_i_ in the hippocampal slices [59]. In the non-treated controls, the vesicle tracks (Figure 4A) indicated the substantial mobility of the Kir4.1-EGFP-positive vesicles; elongated tracks indicate directional mobility and contorted tracks indicate the non-directional mobility (for a detailed description, see [39]) of vesicles (Figure 4A). Already, short-term treatment with KM (2.5 or 25 µM) attenuated vesicle mobility, as indicated by a relatively increased extent of contorted vesicle tracks (Figure 4B). The analysis revealed that vesicle TL was significantly reduced (*p* < 0.05) from 3.46 ± 0.07 µm in the controls to 2.68 ± 0.03 µm and 2.53 ± 0.02 µm in the astrocytes treated for 30 min with 2.5 or 25 µM KM, respectively, and to 2.88 ± 0.02 µm and 2.75 ± 0.03 µm in astrocytes treated for 24 h with 1 mM dbcAMP or 15 mM K^+^ added to the culture media, respectively (Figure 4C). Similarly, the MD was significantly reduced (*p* < 0.05) from 0.87 ± 0.04 µm in the controls to 0.35 ± 0.01 µm and 0.32 ± 0.01 µm in the astrocytes treated for 30 min with 2.5 or 25 µM KM, respectively, and to 0.36 ± 0.01 µm and 0.36 ± 0.01 µm in the astrocytes treated for 24 h with 1 mM dbcAMP or 15 mM K^+^, respectively (Figure 4D). Correspondingly, the vesicle DI was significantly reduced (*p* < 0.05) from 0.23 ± 0.01 in the controls to 0.13 ± 0.00 and 0.13 ± 0.00 in the astrocytes treated for 30 min with 2.5 or 25 µM KM, respectively, and to 0.12 ± 0.00 and 0.12 ± 0.00 in the astrocytes treated for 24 h with 1 mM dbcAMP or 15 mM K^+^, respectively (Figure 4E). Notably, the vesicle speed was also significantly reduced (*p* < 0.05) from 0.23 ± 0.00 µm/s in the controls to 0.18 ± 0.00 µm/s and 0.17 ± 0.00 µm/s in the astrocytes treated for 30 min with 2.5 or 25 µM KM, respectively, and to 0.19 ± 0.00 µm/s and 0.18 ± 0.00 µm/s in the astrocytes treated for 24 h with 1 mM dbcAMP or 15 mM K^+^, respectively (Figure 4F). Cell treatment with various pharmacological agents that may increase intracellular [cAMP] attenuated the mobility of Kir4.1-EGFP-positive vesicles to a similar extent (Figure 4).

### 3.5. Reduced Surface Density of Astroglial Kir4.1 by Ketamine

**Figure 5:**
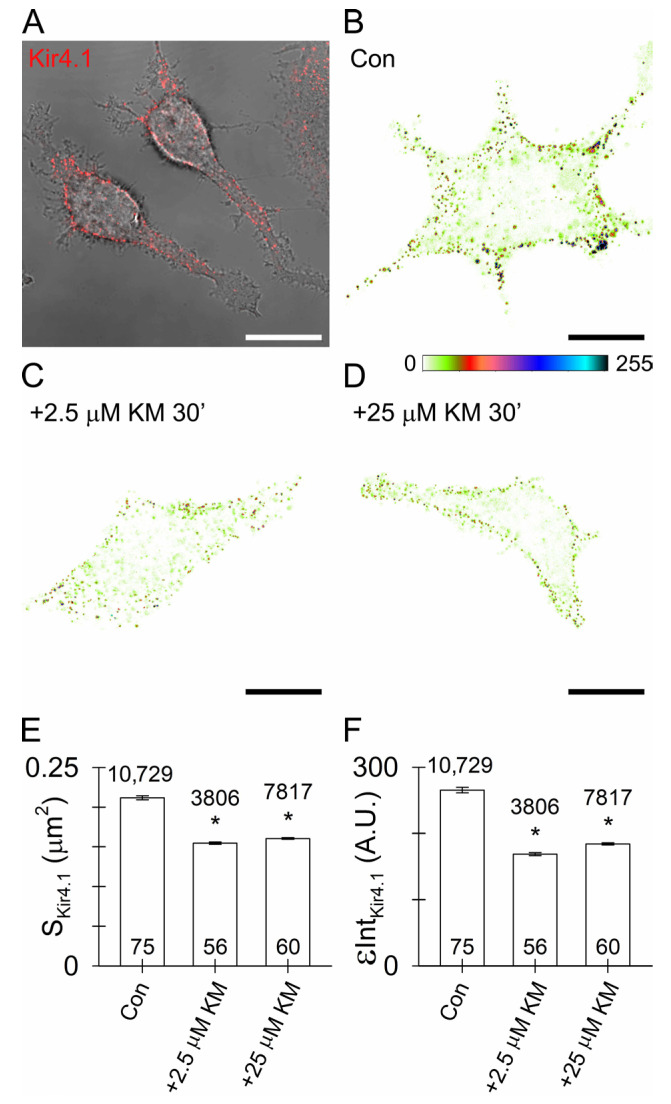
Short-term ketamine (KM) treatment reduced the surface density of Kir4.1 in the cultured mouse astroglia. (**A**) Superimposed transmitted light and confocal image of the immunofluorescent Kir4.1 (labelled by anti-Kir4.1 and by Alexa546-conjugated secondary antibody; red) at the surface of the live mouse astrocytes. (**B**–**D**) Pseudocolored display of immunofluorescent Kir4.1 at the surface of a non-treated control (Con; (**B**)) and the astrocytes treated for 30 min with 2.5 µM (**C**) or 25 µM KM (**D**), respectively. The intensity of Kir4.1 immunolabelling is displayed by the pseudocolored intensity scale (right, 0–255 intensity levels). Scale bars (white, (**A**); black, (**B**–**D**)): 20 µm. (**E**) Surface area and (**F**) cumulative (integrated; ε) intensity (in arbitrary units [A.U.]; mean ± SEM) of the immunofluorescent Kir4.1 at the surface of the non-treated controls (Con) and astrocytes treated for 30 min with 2.5 µM or 25 µM KM. The numbers above and at the bottom of the bars indicate the number of immunofluorescent Kir4.1-positive puncta and the number of cells analyzed, respectively. * *p* < 0.05; ANOVA on the ranks followed by Dunn’s post hoc test versus the surface area (**E**) or cumulative intensity (**F**) of Kir4.1 fluorescence measured in the controls.

KM-evoked reduction in the mobility of Kir4.1-positive vesicles (Figure 4) may in turn reduce the flux of vesicles toward the plasmalemma and hinder incorporation of Kir4.1 by regulated exocytosis [38]. We thus examined whether short-term KM treatment affects the surface density of native Kir4.1 in live mouse astrocytes. Surface Kir4.1 was immunolabelled by the rat primary anti-Kir4.1 and the corresponding fluorescent secondary antibody, as described previously for a different vesicle-delivered molecule [38], in the non-treated controls (Figure 5A,B) and in astrocytes treated for 30 min with 2.5 µM KM (Figure 5C) or 25 µM KM (Figure 5D), respectively. The labelling approach revealed ample Kir4.1-positive immunofluorescent puncta at the surface of the non-treated controls and KM-treated astrocytes (Figure 5A–D), consistent with the enriched Kir4.1 localization in the plasmalemmal microdomains [60] and/or the plasmalemmal folds [61]. However, in the KM-treated astrocytes, the puncta fluorescence appeared less pronounced (Figure 5C,D), which was confirmed by quantifying the puncta surface area and cumulative fluorescent intensity. Short-term KM treatment diminished the area of Kir4.1-positive puncta from 0.21 ± 0.00 µm^2^ in the controls to 0.15 ± 0.00 µm^2^ and 0.16 ± 0.00 µm^2^ in the astrocytes treated for 30 min with 2.5 or 25 µM KM, respectively (Figure 5E). The area of Kir4.1-positive puncta was reduced by 27% and 24% in the KM-treated astrocytes compared with the non-treated controls. The cumulative fluorescence intensity of Kir4.1-positive puncta reduced from 265 ± 4 arbitrary units (A.U.) in the controls to 169 ± 2 A.U. (by 36%) and 184 ± 2 A.U. (by 31%) in the astrocytes treated for 30 min with 2.5 or 25 µM KM, respectively (Figure 5F).

### 3.6. Ketamine Inhibits Voltage-Activated Currents in Astroglia

**Figure 6:**
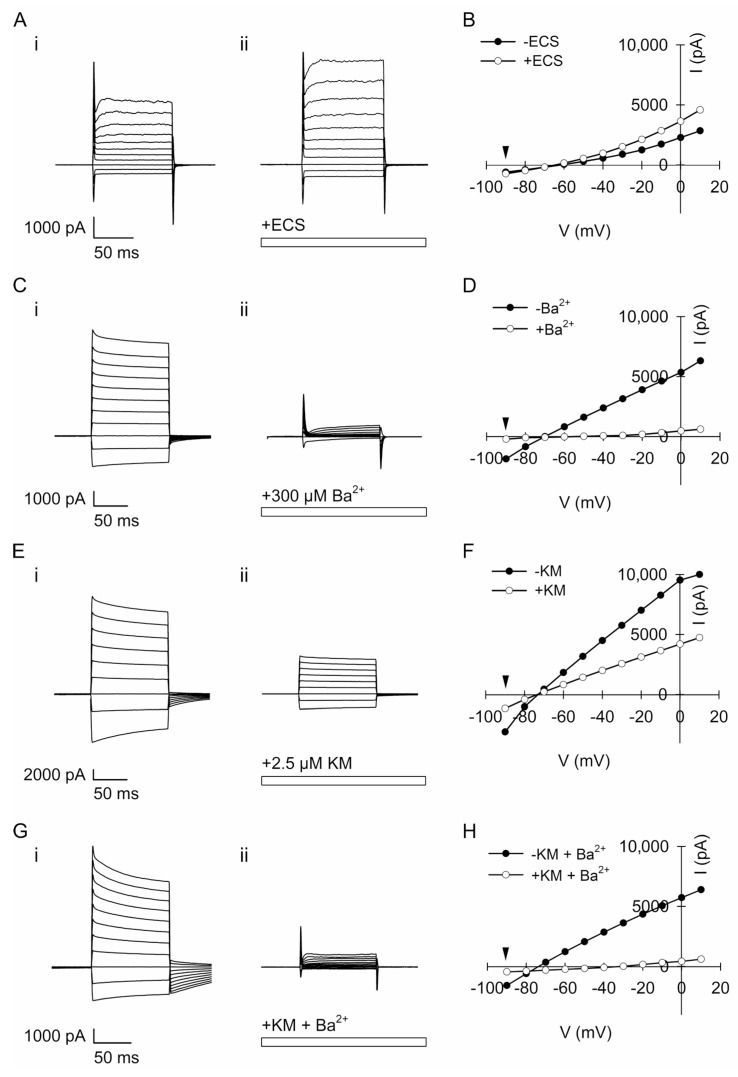
Ketamine and Ba^2+^ treatments inhibit voltage-activated currents in cultured mouse astroglia. (**A**,**C**,**E**,**G**) Superimposed membrane currents evoked by hyper- or depolarized step potentials applied to astrocytes before (**i**) and after (**ii**) acute treatment (up to 3 min) with: ECS (as a control; (**A**)), 300 µM Ba^2+^ (**C**), 2.5 µM KM (**E**), and the corresponding mixture of KM and Ba^2+^ (**G**). Cells were voltage-clamped at −70 mV and hyper- or depolarized in a step-wise manner from −90 mV to +10 mV by 100 ms rectangular pulses in 10-mV increments. The application of ECS or pharmacological inhibitor(s) is indicated by the horizontal rectangle. (**B**,**D**,**F**,**H**) The current–voltage relationship of macroscopic voltage-activated currents (displayed in (**A**,**C**,**E**,**G**)) recorded before (black symbols) and during the treatment (white symbols) with ECS (**B**), 300 µM Ba^2+^ (**D**), 2.5 µM KM (**F**), and the mixture of KM and Ba^2+^ (**H**). Black arrowheads (**B**,**D**,**F**,**H**) indicate the transmembrane potential (−90 mV) at which the current amplitude was measured before and after the application of pharmacological agents (see the Section 2 for details).

To test whether KM affects membrane conductance and astroglial K^+^ currents such as tricyclic antidepressants [62] or selective serotonin reuptake inhibitors [63], we conducted electrophysiological experiments in which mouse astrocytes were voltage-clamped at −70 mV and hyper- or depolarized from −90 mV to +10 mV, before (i) and after (ii) the addition of pharmacological agents to the cells (Figure 6A,C,E,G). Specifically, we measured currents before (i) and after (ii) the application of ECS (Figure 6A), 300 µM Ba^2+^ (Figure 6C), 2.5 µM KM (Figure 6E), or a mixture of KM + Ba^2+^ (Figure 6G) and constructed the corresponding current–voltage relationships (Figure 6B,D,F,H). Because Kir4.1 is heterogeneously expressed in cultured rodent astrocytes [64], we only analyzed currents in cells exhibiting a substantial reduction in the current amplitude upon the application of 300 µM Ba^2+^ (Figure 6C), 2.5 µM KM (Figure 6E), or a mixture of both (Figure 6G).

**Figure 7:**
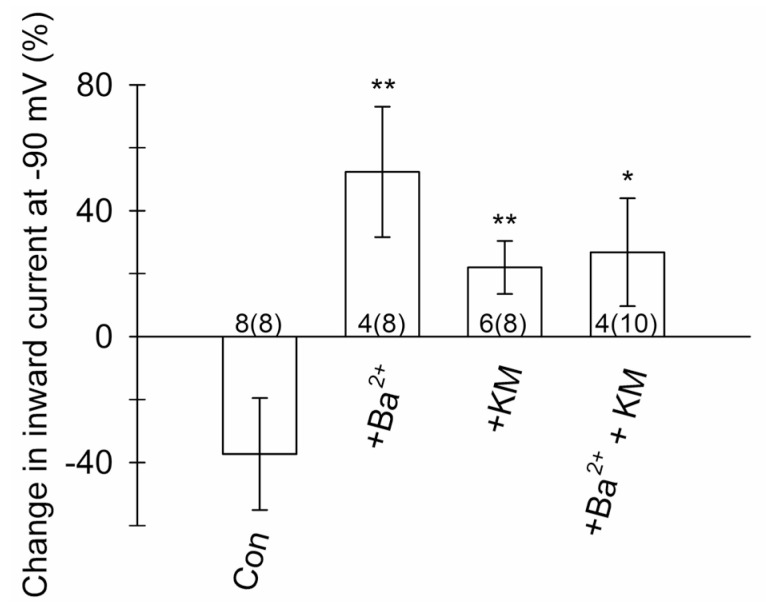
Ketamine (KM) mimics the inhibition of Ba^2+^-sensitive, voltage-activated inward currents in mouse astroglia. Relative reduction (%) in the amplitude of inward (negative) currents (referred as I_K_^+^) measured at −90 mV (calculated as ΔI_K_^+^ = ((I_K_^+^_after_/I_K_^+^_before_) − 1) × 100) before and after (+) cell treatment with ECS (Con), 300 µM Ba^2+^, 2.5 µM KM, and the mixture of KM and Ba^2+^. The numbers at the bottom of the bars indicate the number of cells displaying a reduction in the amplitude of inward (negative) current after the given treatment; the numbers in parentheses indicate the number of all the measured cells. * *p* < 0.05, ** *p* < 0.01 versus control (Mann–Whitney *U* test).

Kir4.1 functions as an intermediate inwardly rectifying channel [65,66] conducting a large inward K^+^ current at membrane potentials negative to the K^+^ equilibrium potential and a small, but significant, outward K^+^ current at potentials positive to the K^+^ equilibrium potential. The analysis of the current amplitude at −90 mV before and after the application of pharmacological agents is shown as the relative reduction (%) in the current amplitude (Figure 7). No reduction in the voltage-activated current amplitude was observed after the application of ECS in the controls; instead, the negative current amplitude tended to increase slightly (eight cells). In cells treated with 300 µM Ba^2+^, the voltage-activated negative current amplitude was reduced by 52.3 ± 20.7% (in four of out eight cells; *p* = 0.008, Mann–Whitney *U* test) compared with the ECS-treated controls. Cell treatment with 2.5 µM KM reduced the voltage-activated negative current amplitude by 21.9 ± 8.4% (in six out of eight cells; *p* = 0.002), whereas treatment with a mixture of both KM and Ba^2+^ reduced the voltage-activated negative current amplitude by 26.8 ± 17.1% (in four out of ten cells; *p* = 0.028).

Thus, our data indicate that a sub-anesthetic dose of KM inhibits astroglial voltage-activated currents that are similarly blocked by Ba^2+^. As further revealed by the analysis of current reversal potentials (Table 1) measured in cells subjected to various treatments (ECS, 300 µM Ba^2+^, 2.5 µM KM, or the mixture of KM and Ba^2+^), the currents partially inhibited by KM were largely due to the influx of K^+^.

## 4. Discussion

Accumulating evidence (reviewed by Stenovec et al. [18]) suggests that KM partially exerts its antidepressant effect via astrocytes that modulate synaptic transmission and neuronal excitability [2]. In this study, we examined how KM alters Kir4.1 vesicle mobility, surface density, and membrane conductance in astrocytes, which control [K^+^]_o_ and tune the neuronal action potential firing in different cortical [67] and subcortical brain regions implicated in reward perception and experience (anhedonia) [21,22,68]. Using an in vitro culture model of enriched cortical astrocytes is a simplified, but suitable approach to address the astrocyte specific effect of ketamine on the function of Kir4.1, which is expressed broadly in the cortex and various subcortical brain regions [23].

### 4.1. Kir4.1 and AQP4 Coalesce on Vesicles Competent for Regulated Exocytosis

In transfected astrocytes, fluorescent Kir4.1 channels localized to the same vesicles as AQP4 (~65%; Figure 1), the predominant channel type involved in water transport in (patho)physiologic conditions [69]. High co-localization of both channels was also reported in end-feet membranes contacting brain micro-vessels and sub-arachnoidal space in brain astrocytes and Müller cells, indicating that spatial K^+^ buffering likely couples with water movement across the astroglial plasma membrane [44,69,70]. As evident from our work, both channels not only co-localize at the surface but are also apparently delivered there by the same vesicles (Figure 1). The co-localization of vesicular Kir4.1-EGFP with SytIV, a positive regulator of astroglial exocytosis [47], and vSNARE VAMP3, indicated that ~1/3 of Kir4.1 vesicles may engage in regulated SNARE complex-dependent exocytosis [71,72]. Psychoactive drugs such as KM affect SytIV expression in the brain [73,74], and SytIV has also been observed to influence exocytotic fusion pore properties [75,76]. Minute co-localization of Kir4.1-EGFP with LC3-positive autophagosomes and subcellular compartments carrying Rab7, which regulates transport from early to late endo-/lysosomes and plays a role in autophagosome maturation [51], indicated that autophagosomes are unlikely to act as vesicular carriers of Kir4.1 in our experiments. Negligible Kir4.1-EGFP co-localization with LyTR-laden acidified late endo-lysosomes (Figure 1) also indicated that Kir4.1 is unlikely to segregate to endo-lysosomes. Because the majority (~57%) of Kir4.1-EGFP vesicles localized in close proximity to the microtubules whereas a minority (~26%) close to the actin filaments (Figure 3C), microtubules may predominantly serve as tracks enabling the directional mobility of Kir4.1 vesicles.

### 4.2. Ketamine Attenuates the Mobility of Kir4.1 Vesicles via a cAMP-Dependent Mechanism

**Figure A1:**
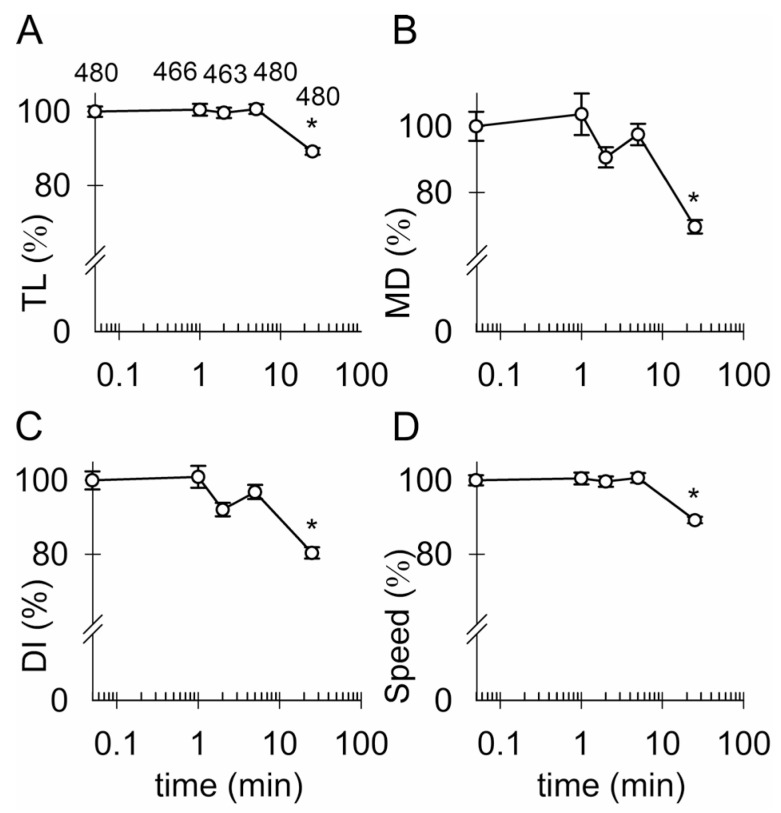
An acute increase in extracellular K^+^ evokes the delayed attenuation of the mobility of astroglial Kir4.1 vesicles. (**A**–**D**) Relative change (in %) in Kir4.1 vesicle mobility (track length [TL], (**A**); maximal displacement [MD], (**B**); directionality index [DI], (**C**); speed, (**D**); %, mean ± SEM) before (0), and 1, 2, 5, and 25 min after the application of high [K^+^] ECS. Note the delayed attenuation of vesicle mobility in response to an acute increase in extracellular [K^+^]. The numbers at the top of the data points in (**A**) indicate the number of 15 s vesicle epochs analyzed in six cells. * *p* < 0.05 versus mobility before the application of a high [K^+^] solution (ANOVA on the ranks followed by Dunn’s post hoc test).

Studies on KM-evoked alterations of vesicle trafficking and the underlying mechanisms have only been recently undertaken [40]. We hypothesize that the mobility of astroglial Kir4.1 vesicles is regulated by a KM-evoked increase in [cAMP]_i_ [32]. Short-term treatment with KM as well as prolonged exposure to dbcAMP and [K^+^]_o_ attenuated the mobility of Kir4.1 vesicles (Figure 4 and Figure A1). A cAMP analog, dbcAMP, slowly permeates through the plasmalemma and hydrolyzes intracellularly to the active form [55,56], but increased [K^+^]_o_ in conjunction with sufficient extracellular HCO_3_^−^ entering via the electrogenic NaHCO_3_ co-transporter may activate soluble adenylate cyclase (sAC) to increase [cAMP]_i_ [57,58,77]. In vivo, an increase in astroglial intracellular HCO_3_^−^ occurs in response to an increase in [K^+^]_o_ caused by increased neuronal activity [78,79]. An increase in astroglial [cAMP]_i_ can also be evoked in response to lactate, fatty acids, aspirin, and antidepressants [56,58]. KM is thought to amplify adrenergic receptor-mediated cAMP signaling, as was demonstrated in C6 glioma cells [80], whereby G_αs_-proteins were translocated from lipid rafts to non-raft-regions, allowing them to interact with and activate adenylate cyclase (AC), increasing cAMP production, even in the absence of G-protein receptor stimulation. A similar effect was observed in primary astrocyte cultures [32]. In C6 glioma cells, the translocation of G_αs_-proteins from lipid rafts to non-raft regions after treatment with 10 µM KM for 15 min was stable for about 24 h before returning to the baseline [80]. Rapid KM-evoked G_αs_-protein translocation in the astrocytes (within minutes) potentially precedes a rapid antidepressant action of KM (1–2 h) in humans [81,82].

How does increased [cAMP]_i_ attenuate the mobility of Kir4.1 vesicles? AC is an enzyme closely involved in cAMP production and associates with G-protein-coupled receptors (GPCRs). In addition to GPCR-associated ACs, astrocytes also express soluble AC [58]. Protein kinase A (PKA), the major intracellular enzyme regulating the activity of GPCRs, is activated by cAMP [83]. However, in B16/F10 murine pigment cells, an increase in [cAMP]_i_ through a PKA-independent mechanism inhibits protein kinase B (PKB, also known as Akt) activity, which regulates the activity of glycogen synthase kinase 3β (GSK3β). If similar processes are present in the astrocytes, an increase in [cAMP]_i_ may decrease the phosphorylation of GSK3β and stimulate its activity [84]. Activation of GSK3β in turn increases the phosphorylation of kinesin, favoring reduced binding of the membrane-bound cargo to the motor protein [85,86]. In vivo, GSK3 phosphorylates kinesin light chains, causing the release of membranous organelles from kinesin-1 and reducing kinesin-1 driven motility [87]. With increased GSK3β activity, the relative level of kinesin light chain phosphorylation increases, whereas the amount of kinesin-1 bound to membranous organelles decreases. GSK3β activity is further affected by calmodulin-dependent kinases (such as CaMKIII), which on binding of Ca^2+^, activates GSK3β, which subsequently phosphorylates molecular motors and inhibits the intracellular transport of brain-derived neurotrophic factor (BDNF) vesicles [88]. In addition, astrocyte treatment with 1 mM 8Br-cAMP for 8 days altered the expression of a wide range of genes including genes important for cytoskeleton organization and function [89]. This differential gene expression could further explain the long-term effect of [cAMP]_i_ on vesicle mobility.

### 4.3. Ketamine Reduces Astroglial Kir4.1 Surface Density

Short-term KM treatment reduced immunofluorescent Kir4.1 labelling (Figure 5), suggesting a reduced surface density of Kir4.1. Insufficient incorporation of Kir4.1 into the plasmalemma may be due to attenuated vesicle mobility (Figure 4). Because the surface density of Kir4.1 was reduced relatively rapidly after the onset of KM treatment, one cannot exclude the possibility that KM directly affects microtubule-associated (Figure 3) motor protein driven mobility. In support of this scenario, Bensel et al. [90] demonstrated that general anesthetics including KM specifically bind to kinesins and/or the kinesin–β-tubulin interface, and inhibit their ability to transport cargo. KM apparently associates with the allosteric binding sites that form transiently in kinesin when the motor domain binds to the microtubule lattice during stepping, and reduces kinesin microtubule affinity. This scenario implies the existence of a druggable allosteric binding site that, when occupied, promotes the detachment of the kinesin motor from the microtubule, leading to reduced run length (total distance travelled per microtubule encounter). Multiple transient binding sites may exist on the kinesin motor domain, requiring only KM binding to the kinesin to have an impact on the interaction between the motor protein and the microtubule [90].

Kir 4.1 also contains archetypal type I PDZ binding motifs (PDZ refers to the initials of the first three proteins discovered to share the domain: PSD-95/DL/ZO-1) [91]. PDZ proteins often serve as anchors for their PDZ binding partners on specific membrane domains. Binding between Kir4.1 and the PDZ proteins can be regulated. In renal tubules, when [cAMP]_i_ is increased, PKA phosphorylation of the critical serine in the type I binding motif of Kir4.1 interferes with the PDZ protein interaction, leading to the unstable anchoring of Kir4.1 to the basolateral surface of renal tubules and the subsequent removal of Kir4.1 [92,93]. Astrocytes also possess proteins with the PDZ domain, the syntrophins; these are found as part of a multiprotein complex known as the dystrophin–glycoprotein complex (DGC). Synthropins are thought to be responsible for binding Kir 4.1 as well as AQP4 to the DGC and enable both channels to be part of the same complex on the plasmalemma [91]. As astrocytes have the prerequisites in terms of required cellular structures/mechanisms (PDZ binding-partners on the plasmalemma, Kir4.1 and PKA), it can be speculated that a similar mechanism, which disrupts the interaction of Kir4.1 with the plasma membrane in renal tubules when [cAMP]_i_ is increased, also exists in astroglia. KM directly increases [cAMP]_i_ [32], therefore, this mechanism could potentially contribute to the decrease in the Kir4.1 surface density. Thus, diverse, but not mutually exclusive mechanisms, may act to reduce the surface density of Kir4.1.

As we conducted our study on astrocytes cultured in medium supplemented with added serum [94], which may be present in the extracellular space under pathologic [95], but not under normal conditions, one may ask whether the results regarding the Kir4.1 surface expression may be affected by the experimental conditions (i.e., reactive astrocytosis) as reactive astrocytes may differentially express Kir4.1 [96]. However, substantial alteration of Kir4.1 expression in an astrocyte-enriched culture appears unlikely, since electrophysiological measurements of the resting membrane potential, the negativity of which is determined by Kir4.1 channels [64], in the cultured cortical rat astrocytes enriched by the shaking procedure [97] and in freshly isolated rat hippocampal astrocytes revealed similar bimodal resting membrane potential distributions [98]. Moreover, cultured cortical rat astrocytes enriched by an alternative, non-shaking, procedure [64] also displayed bimodal distribution of the resting membrane potentials with peaks at −68 mV and at −41 mV, with 77% of cells predominantly in the hyperpolarized population [64]. Importantly, no correlation was found between the morphology of the cultured astrocytes and resting membrane potentials, as both flat and arborized astrocytes displayed bimodal distribution of the resting membrane potentials with peaks at −65 and −43 mV (arborized astrocytes) and −69 and −45 mV (flat astrocytes) [97]. In the cultured astrocytes, the resting membrane potentials were affected only by the knock-down of Kir4.1 channels, after which astrocytes with hyperpolarized potentials were mostly absent, whilst the remaining resting potentials peaked at −45 mV [64].

### 4.4. Ketamine Inhibits Astroglial K^+^ Conductance

Antidepressants, particularly tricyclic antidepressants and selective serotonin reuptake inhibitors, may interact directly with the Kir4.1 channel [99] to inhibit K^+^ currents [62,63]. Inhibition of Kir4.1 affects neuronal excitability [21] and presumably underlies an antidepressant effect [100] because altered K^+^ siphoning favors an increase in extracellular [K^+^], reduces the clearance of extracellular glutamate at the synapses [100], and facilitates the expression of BDNF in astrocytes [101]. Unexpectedly, KM also inhibited Ba^2+^-sensitive voltage-activated K^+^ currents in the astrocytes (Figure 6 and Figure 7). This finding is important because the upregulation of astroglial Kir4.1 in LHb, a tiny region in the diencephalon [102], shifts the pattern of neuronal activity from regular to burst firing, leading to an inhibition of the downstream reward centers and resulting in depressive-like states such as behavioral despair and pleasure deficit [21].

How is the Kir4.1-mediated K^+^ current inhibited by KM? The phosphorylation of ion channels by protein kinases is a way to regulate membrane excitability in cells [103], and the cAMP/protein kinase A (PKA) cascade is considered as an important regulator of depression because it affects the K^+^ currents that regulate neuronal excitation [104]. Protein kinases such as PKA and protein kinase C (PKC) regulate Kir activity by the phosphorylation of channel subunits. In cells stimulated via G-protein (G_s_)-coupled receptors, Kir6.2 was phosphorylated by PKA at its C-terminal serine 372 phosphorylation site [105]. A typical PKA phosphorylation site was also found at serine 430 on Kir2.2 [106]. Both studies suggest that PKA regulates K^+^ channels by directly phosphorylating Kir. In Kir4.1, there are sites that can be phosphorylated, and Src family protein tyrosine kinases regulate the basolateral K^+^ channels in the distal convoluted tubule through Kir4.1 (KCNJ10) phosphorylation [106]. Moreover, the C terminus of Kir4.1 and Kir5.1 contains sites that can be directly phosphorylated by PKC, whereas the activation of PKC can inhibit the opening of Kir4.1-Kir5.1 channels in HEK293 cells [107]. Collectively, these studies suggest that PKA may phosphorylate Kir4.1 and inhibit Kir4.1 channel activity to alter astroglial K^+^-spatial buffering [62]. Notably, KM-induced changes in the astrocyte plasmalemmal structure may activate adenylyl cyclase and increase [cAMP]_i_ within minutes [32].

As an N-methyl-D-aspartate-receptor complex [108,109], KM potentially directly binds to Kir4.1 and inhibits the K^+^ current [99] either from the extracellular or intracellular side after first permeating into the cells. When applied externally, KM, a weakly basic arylcycloalkylamine with a pK_a_ of 7.5 [109], easily crosses the plasmalemma and accumulates inside acidified vesicles in the protonated form [110]. However, this route of KM action is unlikely because Kir4.1 does not segregate in acidified vesicles and is not delivered to the surface by these organelles (Figure 1 and Figure 3). It is also unlikely that non-protonated KM enters the selective filter of K^+^ channels [111]. The diameters of Ba^2+^ (2.68 Å; a permeant blocker of K^+^ channels) [111] and KM (estimated 7.07–11.35 Å [112]) differ substantially, therefore, it is unlikely that KM blocks the K^+^ channel at the same spot along the pore as Ba^2+^. Future work is required to clarify the mechanism of Kir4.1 inhibition by KM.

## 5. Conclusions

Here, we provide an immunocytochemical characterization of astroglial Kir4.1 vesicles and demonstrate that cAMP-dependent attenuation of Kir4.1 vesicle mobility diminished astroglial Kir4.1 surface density and the inhibition of voltage-activated inward K^+^ currents. These alterations may contribute to altered astroglial K^+^ buffering, possibly underlying an antidepressant effect in a rat model of depression [21]. We conclude that KM exerts pleiotropic effects on astroglial Kir4.1 that may contribute to antidepressant effects. Understanding astroglial mechanisms of KM action might help facilitate the development of novel astrocyte-targeted antidepressants with minimized potential for abuse and the induction of a transient psychotic state.

## Figures and Tables

**Table 1:** The reversal potential of the voltage-activated currents measured in the cultured mouse astrocytes treated with various pharmacological agents.

Treatment	Inhibited (All) Cells	E_rev_ (mV)−	E_rev_ (mV)+	*p*-Value
ECS (control)	8 (8)	−64 ± 3	−57 ± 3	0.130
300 µM Ba^2+^	4 (8)	−53 ± 7	−53 ± 5	0.886
2.5 µM KM	6 (8)	−65 ± 7	−63 ± 7	0.589
KM + Ba^2+^	4 (10)	−73 ± 0	−60 ± 10	0.029 *

The reversal potential (in mV) of the voltage-activated K^+^ currents measured in the astrocytes before (−) and after (+) treatment with ECS, 300 µM Ba^2+^, 2.5 µM KM, and the mixture of KM and Ba^2+^ (mean ± SEM). * *p* < 0.05 is significant (Mann–Whitney *U* test, − vs. +).

## Data Availability

The datasets supporting the current study have not been deposited in a public repository but are available upon request from the corresponding author.

## Appendix A. An Acute Increase in [K+]o Requires a Delay to Alter the Mobility of Kir4.1 Vesicles

We also examined whether an acute, short-term increase in [K^+^]_o_ altered the mobility of the Kir4.1 vesicles. The mobility of the Kir4.1 vesicles was not immediately affected by an acute increase in [K^+^]_o_ from 5 to 15 mM at 1, 2, or 5 min after the addition of high [K^+^] ECS. The mobility of the Kir4.1 vesicles decreased only 25 min after the addition of high [K^+^] ECS (*p* < 0.05; Figure A1A–D). A pathophysiologically relevant increase in [K^+^]_o_ apparently requires a delay to affect the mobility of Kir4.1 vesicles.
